# Preventing falls: the use of machine learning for the prediction of future falls in individuals without history of fall

**DOI:** 10.1007/s00415-022-11251-3

**Published:** 2022-07-11

**Authors:** Ioannis Bargiotas, Danping Wang, Juan Mantilla, Flavien Quijoux, Albane Moreau, Catherine Vidal, Remi Barrois, Alice Nicolai, Julien Audiffren, Christophe Labourdette, François Bertin‐Hugaul, Laurent Oudre, Stephane Buffat, Alain Yelnik, Damien Ricard, Nicolas Vayatis, Pierre-Paul Vidal

**Affiliations:** 1 Centre Borelli, CNRS, SSA, INSERM, Université Paris Saclay, Université Paris Cité, ENS Paris Saclay, Gif-sur-Yvette, 91190 France; 2 Centre Borelli, CNRS, SSA, INSERM, Université Paris Cité, Université Paris Saclay, ENS Paris Saclay, Paris, 75006 France; 3 ORPEA Group, Puteaux, France; 4 Service of Otorhinolaryngology (ENT), AP-HP, Hôpital Universitaire Pitié Salpêtrière, Paris, 75013 France; 5 Department of Neuroscience, University of Fribourg, Fribourg, Switzerland; 6 Laboratoire d’accidentologie de biomécanique et du comportement des conducteurs, GIE Psa Renault Groupes, Nanterre, France; 7 Service of Physical and Rehabilitation Medicine (PRM), AP- HP, GH St Louis, Lariboisière, F. Widal, Paris, 75010 France; 8 Service of Neurology, AP-HP, Hôpital d’Instruction des Armées de Percy, Service de Santé des Armées, Clamart, 92140 France; 9 École d’application du Val‐de‐Grâce, Service de Santé des Armée, Paris, France; 10 Institute of Information and Control, Hangzhou Dianzi University, Zhejiang, China

**Keywords:** Fall prediction, Frailty, Machine learning, Wearables, Force-platform, Longitudinal follow-up

## Abstract

Nowadays, it becomes of paramount societal importance to support many frail-prone groups in our society (elderly, patients with neurodegenerative diseases, etc.) to remain socially and physically active, maintain their quality of life, and avoid their loss of autonomy. Once older people enter the prefrail stage, they are already likely to experience falls whose consequences may accelerate the deterioration of their quality of life (injuries, fear of falling, reduction of physical activity). In that context, detecting frailty and high risk of fall at an early stage is the first line of defense against the detrimental consequences of fall. The second line of defense would be to develop original protocols to detect future fallers before any fall occur. This paper briefly summarizes the current advancements and perspectives that may arise from the combination of affordable and easy-to-use non-wearable systems (force platforms, 3D tracking motion systems), wearable systems (accelerometers, gyroscopes, inertial measurement units-IMUs) with appropriate machine learning analytics, as well as the efforts to address these challenges.

## Scientific context

One of the major causes of injury in the elderly (and also in pathological cases such as Parkinson’s disease [1], Dementia [2], and many others) is falling, resulting in further mobility restriction, diminution of functional ability, an increase of caregiver burden, autonomy problems in daily activities (bathing, cooking, etc.) or even death. Falls are not only the second leading cause of accidental death; they are also a significant source of stress for the elderly [3, 4]. Almost 30% of the population > 65 years old [5] and 60% of the patients who have Parkinson’s disease [1] face at least one fall per year. According to these numbers, the accurate evaluation of the risk of falling (the sooner, the better) through predictive methodologies is crucial. Moreover, fear of falling again, even after falls that did not require medical treatment, is associated with a vicious circle of further avoidance of activities of daily living, less physical activity, multiple fallings, depression, and lower quality of life [6].

The causes of falls are multifactorial (medication use, complex activities, stress, environmental complexities, and sleep quality) [7–9]. In addition, individuals that enter in a pre-frail or frail condition experience deterioration and de-harmonization in neuromuscular, sensory, and cognitive functions. Finally, considering the fact that static postural control [10], locomotion, and navigation are tasks that have high cognitive demands, especially in terms of attention [11] as well as executive functioning [12, 13], deterioration at the sensorimotor and cognitive levels leads to gait and balance disorders increasing the risk of fall [1, 2, 8, 10–15].

In this context, it is not surprising that over the past 15 years, numerous studies have been devoted to the prevention of falls. However, given the abundance of literature on the subject, the next section will only cite the reviews devoted to this subject.

### Prediction of fall: current knowledge

We should make several preliminary points: first, fall prediction mainly focuses on people who have not fallen yet. We know that every elder adult who falls once will probably fall again [16]. Also, it should be remembered that walking speed is a predictor of survival in the elderly [17] (See Mobilise-D initiative outcomes^1^) and conversely a good indicator of future falls. Finally, fighting sedentary lifestyle, sports practice, and exercise programs designed to prevent falls in older adults prevent injuries caused by falls, including the most severe ones, and reduce the rate of falls leading to medical care [18]. With these preliminary remarks, a first point is a consensus for at least a decade about the inadequacy of clinical tests to assess future fall risk alone. As early as 2012, Roza da Costa et al. [19] concluded that they could not identify any tool with an optimal balance of sensitivity and specificity or that was better than a simple clinical judgment of fall risk. The tools tested consisted of the STRATIFY tool, the PJC-FRAT, the DOWNTON Fall Risk Index, and “clinical judgment” [20]. Recently, Omana et al. [21] sought to systematically review the existing literature on the properties of the fall-related diagnostic tests, namely the Functional Reach Test (FRT), the Single Leg Stance Test (SLST), and Tinetti’s Performance-Oriented Mobility Assessment (POMA), in older adults, in different settings and patient populations. They conclude that neither the FRT, SLST, nor POMA alone show consistent evidence of correctly identifying people who fall, regardless of the type of fall, setting, or elderly subpopulation. Lusardi et al. [22] attempted to evaluate the predictive ability of history questions, self-report measures, and performance-based measures to assess fall risk in community-dwelling older adults by calculating and comparing post-test probability values (PoTPs) for individual tests/measures. No test/measure demonstrated high PoTP values. However, they note that five history questions, two self-report measures, and five performance-based measures may have clinical utility in assessing fall risk based on cumulative PoTP. The Berg Balance Scale score (≤ 50 points), timed rise and fall times (≥ 12 s), and fivefold change from sitting to standing (≥ 12) may help determine individual risk of fall. Barry et al. [23] concluded that the Timed Up and Go test has a limited ability to predict falls in community-dwelling older adults and should not be used in isolation to identify individuals at high risk for falls in this setting. They also pointed out the promising added value of dual tasks involving turns and other transfers, as in the Timed Up and Go test, for predicting falls, which will be useful when accelerometers become available a few years later. In summary, these reviews highlight the interest in clinical examinations and underline their inability to predict efficiently future falls in a person who has never fallen.

Therefore, it is understandable that in this context, several tools to measure postural control and locomotion have been developed to prognosticate future falls. Along that line of view, laboratory tests that record the center of pressure (COP) trajectory have been extensively employed. However, despite their widespread use, the choice of COP trajectory features for use as a biomarker of fall risk lacks consensus. A systematic review and meta-analysis of Quijoux et al. [24, 25] aimed to identify the best COP characteristics to predict the risk of falling in older adults. Several COP parameters emerged as good indices to discriminate fallers from non-fallers. From sensitivity analysis, Sway area per unit time, antero-posterior mean velocity, and radial mean velocity were the best standard features. This study demonstrated the identification of older people with a high fall risk using quiet-standing recordings. Such screening would be helpful for routine follow-up of balance changes in older fallers in clinical practice.

Then, two trends can be recognized. Firstly, systems that *detect* a risk of fall (if possible) or a fall occurrence and activate an alarm. Cortes et al. [26] reviewed the use of hospital sensors as an adjuvant in the medical/nursing care plans to alert health care providers about the risk of fall according to their programmed sensitivity and specificity. They included call bells and alarms in the rooms of hospitalized patients to alert caregivers about patients’ needs of movement that demand help, movement, and pressure sensors installed on furniture such as beds or chairs. However, the sensors identified in this meta-analysis failed to predict falls in real-time. Secondly, wearable sensors were used to *predict* and *prevent* falls. In that context, the revolution of the last five years is the availability of sensors that allow for a reasonable price to measure the mobility of people in consultation. In particular, the inertial measurement unit (IMU) is an electronic component that measures the sensor’s acceleration, angular velocity, and orientation using a combination of accelerometers, gyroscopes, and magnetometers. Small size, very reasonable price (about two to 300 € per sensor), several IMUs can easily be strapped to the upper limbs, lower, head and trunk. They communicate via a wireless link to a computer that, equipped with appropriate programs, can instantly provide clinicians with a very accurate measurement of their patients’ mobility. Also, personal devices, such as smartphones, are beginning to be used for implementing fall systems because they are carried by the users most of the day. Ferreira et al. [27] review showed that IMUs were generally placed in the upper body and that machine learning models were preferably adopted to classify the subject’s risk of fall. However, they noted that the number of participants enrolled in the studies they reviewed was often reduced and sometimes did not include elderly participants.

Nevertheless, this review suggested that some fall risk assessment systems obtained an acceptable performance. The authors underlined that an open-access gold standard should be established to allow the benchmarking of different fall risk assessment systems. The latter would pave the way for a reliable performance comparison between the different systems developed in the literature. Interestingly, Hemmatpour et al. [28] reviewed various fall prediction and prevention systems that used machine learning algorithms, particularly those that relied on the sensors embedded in a smartphone, i.e., accelerometer and gyroscope. An experimental analysis compared the evaluated approaches by evaluating their accuracy and ability to predict and prevent a fall. Results show that tilt features combined with a decision tree algorithm present the best performance.

Similarly, Montesinos et al. [29] conducted a systematic review to identify optimal combinations of sensor locations, tasks, and feature categories. The results from their walking test demonstrated that the most compelling feature to assess the risk of fall was the velocity with the sensor placed on the shins. Conversely, linear acceleration measured at the lower back was the most effective combination of feature placement during quiet standing. Similarly, during the sit-to-stand and the stand-to-sit tests, linear acceleration measured at the lower back seems to be the most effective feature-placement combination. In addition, the meta-analysis demonstrated that four features resulted significantly higher in fallers: the root-mean-square acceleration in the medio-lateral direction during quiet standing with eyes closed; the number of steps and total time to complete the Timed Up and Go test; and the step time during walking. Finally, Sun and Sosnoff’s [30] review gave a sober view of sensors to predict the fall. Four major sensing technologies (inertial sensors, video/depth camera, pressure sensing platform, and laser sensing) were reported as capable of providing accurate fall risk diagnostic in older adults. Overall, these technologies have the potential to provide a precise, affordable, and easy-to-implement evaluation of risk-of-fall. However, the variation in assessment tools, measured parameters, sensor sites, movement tasks, and modeling techniques precludes a firm conclusion on their capacity to predict future falls. Future work is needed to determine a clinically meaningful and easy to interpret fall risk diagnosis utilizing sensing technology. In addition, the gap between functional evaluation and user experience with technology should be addressed.

The future prognostic tools should ideally have: (1) the ability to inform the expert about the systems which are involved in the loss of balance (vestibular, visual, proprioceptive), (2) the ability to provide objective and quantified scores, as well as (3) the capacity to provide better information about the future risk of falling, than the history of previous falls [19, 31, 32]. Several tools have been proposed to measure and quantify the risk of fall in community-dwelling older adults. For example, cheap force platforms are now available to monitor static postural control [33–35]. In addition, wearable systems such as accelerometers, gyroscopes or inertial measurement units (IMUs), 3D motion tracking systems, and gaze tracking systems can now be used in clinics for gait analysis and mobility monitoring [36–41].

## Methodological approach and results

### Prediction of falls, machine learning, and modeling

Ideally, the prediction of a fall should consider both physiological and psychological factors. Nowadays, predictive modeling, especially those supposed to have additive value to the clinicians’ daily practice, mainly focuses on physiological risk factors concerning gait, posture, and oculomotor control. As a result of the multiplication of available sensors, there is an explosion in the number of parameters available for clinical research (various biomarkers, multiple modalities, or parameters) for a given cohort of patients. Although beneficial and prosperous, this fact challenges the data analysis via traditional approaches usually met in clinical research (T-tests, standard univariate approaches, etc.). Giving just a simple example, in a classification problem with 20 parameters to be explored if they are associated with a specific phenomenon, 1 in 20 associations may be statistically significant but not clinically meaningful (alpha level *α* = 0.05) [42]. Since traditional statistics become very sensitive when only small multidimensional datasets are available, it is unsafe to make safe generalizations of any finding (see Ref. [43] for the increasing risk of false conclusions). Machine learning algorithms reduce the later limitation, assessing their results using cross-validation schemes. Schematically, an algorithm trains a model in a representative part of the dataset (called training-set) and learns any “rules” that may exist. Then, it tests whether it can be effective in predicting the question of interest (e.g., if an individual is a faller or a non-faller) in the rest of the dataset (test-set: the “unknown” and “unseen” by the algorithm). Repeating multiple times the above process, and keeping track of the performance every time, can give the clinician the ability to evaluate the risk of generalizing these results to a new unknown population (e.g., future patients). With the increasing available computed parameters, the available acquisition systems, and the general trend of fusing information from different modalities, there is a legitimate interest in utilizing modern machine learning algorithms to assess the risk of fall. Recent research on wearable technologies (such as accelerometers and gyroscopes) and force platforms for fall prediction has primarily focused on fusing information and computing parameters for fall risk assessment and applying predictive models to the available datasets [44–49].

In this context, we will present the main trends of machine learning considering the prediction of falls using gait and balance measurements, focusing on pre-frail adults. Although machine learning approaches can offer a qualitative alternative to classical statistical strategies for extracting information concerning falls, they are also more prone to non-interpretable results by the users. Therefore, such approaches should always be justified, and their output should be interpretable and carefully described. Furthermore, final models and algorithms should be assessed by clinical experts to ensure compatibility with clinical practice [50]. Therefore, our objective is to identify solutions that are promising in terms of fall prediction (especially for prospective prediction), and feasible to be applied in daily practice. The following sections are separated by the acquisition modality and describe our effort to tackle these challenges. The proposed machine learning methodologies are distinguished by the broad categorizations of shallow learning and deep learning. Roughly, shallow machine-learning algorithms usually rely on expert-based variables and features. In contrast, deep learning algorithms enable us to a) learn abstract numerical patterns or sequences of patterns from signals or time-series (probably without known clinical explanation) and b) extract useful representations of raw data (inherent feature engineering), to optimize the performance of the fall prediction.

### Fall prediction via posturography

Daily practice of postural control evaluation in community-dwelling elderly populations often uses balance assessment scales. These scales [51–53] do not require any specific training from the operator, and they fit well in the clinical practice. As previously mentioned, the extensive works of Quijoux et al. [24, 25] identified the best COP characteristics to predict the risk of falling in older adults using traditional univariate statistics. From sensitivity analysis, Sway area per unit time, antero-posterior mean velocity, and radial mean velocity were the best standard features. However, features’ effectiveness in predicting future falls in the elderly has been challenged, and researchers recommend cautious interpretation of their results [23, 54].

Additionally, monitoring the individual’s postural control progress using balance assessment scales is not trivial [55]. Recent studies from our lab [47–49, 56–59] and others [60] proposed that a linear and non-linear combination (using machine learning methodologies) of many global or local posturographic parameters derived from the Centre of Pressure (CoP) trajectories can classify fallers and non-fallers. It is important to stress that these algorithms can evaluate the risk of fall in individuals who had not experienced any fall before the acquisition on the force platform (standing eyes open and eyes closed for 30 s each time). Our study showed that the shape of the separating rule (or decision surface) between fallers and non-fallers lies in a multivariate space which is only detectable when all features (computed parameters) are participating ensemble in the learning process (not one by one). Generalizing a retrospective classification of fallers and non-fallers leads us to pick the variables allowing the prospective prediction of future falling.

Practically speaking, there are numerous efforts toward using shallow learning algorithms, using features calculated from posturographic trajectories. The objective is usually dual. On the one hand, create a predictive model that can score the risk of fall and highlight those posturographic features that contribute more to this prediction. For example, Nicolai et al. [56] introduced a *shallow learning* classifier (called H-bagging) to evaluate the risk of falling in the elderly using machine learning and features calculated from CoP trajectories using the posturographic data of the Romberg test. They reported strong performances in healthy populations while producing models that are easy to understand and interpret. Briefly, H-bagging trains multiple univariate classifiers (one per feature) and searches for the best combinations to aggregate their learning decision. They proved that such a strategy significantly increases the performance of the learning process.

Investigating the importance of the non-linear association between fall and posturographic features from the Romberg test, Audiffren et al. [47] classified relatively accurately fallers and non-fallers by implementing Ranking Forest. The ranking forest is a shallow-learning classifier that aggregates the decision rule of multiple ranking trees to create a general decision rule (see Ref. [61] for details). This work proved that, on the one hand, the community was right till then, reporting that the Romberg test does not contain fall-related elements. None of the utilized features alone could classify effectively elderly fallers/non-fallers (i.e., weak classifiers) alone. On the other hand, they proved that an optimal combination of those weak classifiers through non-linear multidimensional classification gave significantly better results in an elderly population, showing the beneficial effect that machine learning can have on such questions. This study also revealed the features with the higher predictive power in terms of risk of fall.

The studies of Speiser et al. [50], Eichler et al. [62], Bargiotas et al. [48, 57, 59], Su et al. [60], Liu et al. [63] also used shallow learning classifiers and most of them the Random forest (RF) [64] that, similar to the work of Audiffren et al. [47], aggregates the decision rules of several decision trees, to evaluate the risk of falling. Su et al. [60] proposed a more direct predictive modeling with encouraging results, reporting the importance of every feature in evaluating risk. Bargiotas et al. [48, 59], proposed an effective alternative to two-sample hypothesis testing (T-tests etc.) for multidimensional datasets to reveal the posturographic features with the higher predictive power in terms of risk of fall. Random forest was also used in a study [57] where a *prospective* prediction of fall is proposed using the differences between features derived by the trajectories of the two protocols of the Romberg test (eyes closed–eyes open). The learning process was completed in the dataset of the first acquisition and tested in the follow-up acquisition six months later.

Most of the aforementioned machine-learning approaches evaluated the risk of fall using features calculated from the whole posturographic trajectory (homogeneity assumption). Despite their significant usefulness, these approaches could not provide information about each trajectory’s interesting parts (time-blocks with interesting oscillations). Similarly to previous works (from signal processing point of view [65]), It was hypothesized that signal blocks (time-blocks) with significantly different properties might co-exist in every individual [49](they separated them into quiet and unquiet blocks—QBs and UBs). Fallers will have significantly different QB/UB combinations than non-fallers. They trajectories’ time-blocks were grouped with a shallow soft unsupervised classification in two clusters (UBs/QBs) based on the Expectation–Maximization algorithm (EM) for Gaussian Mixture Models (GMM). After the reunification of the blocks, the trajectories could provide the individual’s risk of fall and interesting trajectory visualization [58].

With the recent advancement in neural networks, deep learning started to play a dynamic role in assessing the risk of fall. In 2021, Savadkoohi et al. [66] proposed a one-dimensional convolutional Neural network (CNN) trained in posturographic trajectories to predict the outcomes from a questionnaire about the fear of falling (the Falls Efficacy Scale (FES) score). FES was separated into three groups (low, moderate, and high fear of falling). Furthermore, they avoided extracting features from the CP trajectories and classified the force-plate balance time series directly to predict human balance impairment.

Nicolai et al. [67] introduced a new Langevin-based model, called local recall, that integrates the information from both the center of pressure (CoP) and the center of mass (CoM) trajectories to predict the CoP trajectories of an individual. This work further extended the understanding of postural control’s underlying local and global mechanisms during quiet stance. Except for the damping force and a Brownian noise, this new model introduces a recall force that pulls the CoP position toward the CoM position instead of the center of the base of support. It was shown (in multiple datasets) that such choice significantly improves CoP trajectory predictions compared to a commonly used Langevin model. In addition, the model also calculates the relative importance of each force, which may improve the understanding of several aspects of postural control and the differences between examined populations or acquisition protocols.

The above advancements showed the beneficial effect of machine learning on posturography analysis, especially on fall prediction. Recently, our lab published an extensive review [25] concerning the calculation methods of the features derived by the posturographic trajectories of CoP. On the one hand, this review highlighted the lack of homogeneity and standardization between research works and, on the other hand, presented a comprehensive compendium of exact calculation methods for every proposed feature. This review also offers a corresponding python library to facilitate future researchers in their hypothesis, the standardization, the comparison between studies, and the progress of posturography and its analytical approaches.

### Gait analysis, sensors, analytics, and future challenges

Over the past years, our group has done extensive research on the quantification of locomotion, especially in the validation and the preparation of the datasets required to use machine learning algorithms and the prediction of falls. For gait analysis, reasons and details about the decision of one protocol or another are often missing. They sometimes may depend on pragmatic factors such as the price of sensors and the available setting (laboratory or clinical). Although the current clinical practices such as time Up and Go (TUG) [68] or Short Physical Performance Battery (SPPB) are accessible and are used to identify gait alterations, their ability to draw firm conclusions regarding the prediction of fall has been recently questioned [23, 69]. These tests have also been reported as less sensitive in detecting subtle gait dysfunctions in community-dwelling populations or accurately and objectively follow-up progressive (drifting) deteriorations [70]. However, considering this fact, we and others recently highlighted the promising perspectives that the use of IMUs may have in neurological practice (in terms of cost, precision, validity, and information). It was shown that with appropriate setting and analytics, IMUs measurements, composed of accelerometers, gyroscopes, and magnetometers, can detect the progression of gait alteration in the elderly and patients with neurological diseases such as Multiple sclerosis [71, 72]). In addition, gait parameters such as step length, step duration, or walking speed, easily calculated with IMUs, can facilitate the follow-up of a disease or aging-related deterioration [38].

However, we also noticed the need for better further homogenization of protocols to facilitate the validation of normative values per case and the comparison of the manifestations of different diseases [38]. Among the gait features that have been found associated with gait dysfunctions, many of them are related to the notion of step (step duration, step length, variation of step length) [73]. Hence, one of the significant challenges of using IMUs, before evaluating the risk of fall is the accurate detection of steps that pose several problems. A significant challenge is the applicability of the algorithms in various populations with different styles of locomotion and the detection of steps when neurological diseases alter gait. Another challenge is the description of U-turns, turns, gait initiation, or gait termination, which are very frequent in daily activities and elements of paramount importance in fall prediction [74, 75]. Previous works [76] proposed the notion of “template” to detect the steps of a specific population. A template can be considered a typical step whose characteristics (amplitude, shape, and duration) can be the same in all steps. Therefore, almost by definition, a template for one population is not the most appropriate approach to detect the steps in heterogeneous cohorts. Instead of trying to detect steps with one specific template [76, 77] or with traditional filtering/thresholding/peak detection methods, recent works [78, 79] proposed the use of a library of templates that represent typical step cycles from many different populations (young, elderly, pathological). Their results showed that using such a methodology (based on a greedy shallow machine learning algorithm and a library of annotated step templates) improved the robustness of the detection, even with a small number of templates. Performance in an extensive database with mixed populations (healthy, pathological, young, elderly) with different walking characteristics was 98% recall and 98% precision.

Moreover, the algorithm detected starting and termination points of each step in U-turns (83.87% recall and 90.76% precision) which is a significant amelioration of state of the art, even on pathological subjects. One of the additive values of this approach is that it can be easily extended to process signals recorded in free-living conditions and use fewer IMUs sensors (e.g., only waist signals). Indeed, having libraries for no activity and U-turns facilitates the adaptation of the proposed method to unconstrained walking. For the interested reader, we have recently used the concept of the template to study locomotion in different populations [80].

Gait research counts numerous well-established spatio-temporal gait parameters, which can provide interpretable information and help modern models to achieve higher performances. Recent works placed wearable sensors at the lower back [81], ankles, hip [82] or waist [83] and calculated various Spatio-temporal gait variables while 20 m-walking. They managed to discriminate between fallers and non-fallers using shallow learning (SVM [82, 83], Partial least square discriminant analysis (PLS-DA) [81]) and improve our understanding of how falls-related gait impairments in neurological patients are manifested. Similarly, [84] used Spatio-temporal variables from shoes’ IMU sensors during 20 m walking and shallow learning (extreme gradient boosting (XGBoost), a decision tree-based ensemble machine learning technique). They found that stride length and walking speed are the most important variables regarding future risk of fall. XGBoost algorithm was also used for future fall prediction, using demographic and individuals’ medical profiles [85].

Other studies [46, 86] successfully applied deep learning using similar accelerometer positions. Specifically, the study in [46] uses convolutional and bidirectional long short-term memory layers, to learn from spatiotemporal features from 2 wrist-worn IMUs. They manage to discriminate regular 15 m-walking sessions from the “distorted” ones (walking with impairment glasses). As we mentioned previously, deep learning has the potential to produce models that can learn directly from the IMUs time-series, thus alleviating the need for feature extraction and selection. Other approaches [87] combined information from time-series and established spatio-temporal variables creating a deep learning model for the problem of fall risk assessment. They utilize sequences of spatio-temporal gait parameters extracted by an inertial sensor-based gait analysis system as input features showing improved performance compared to the models that did not utilize combined information.

### Open and reproducible research

Surprisingly, compared to many published articles concerning gait and IMUs, there are very few datasets that are, on the one hand, freely available and, on the other hand, documented enough to be used for further research. This lack of open and documented datasets does not allow the clinicians to test (in a reproducible manner) new clinical hypotheses such as the discriminative power of walking patterns in a faller/non-faller population [88]. Moreover, there are no opportunities for mathematicians and biomedical engineers to design new algorithms, compare various frameworks and reach consensus in terms of the appropriate analytic approaches. Some initiatives are promoting the above objectives and including gait signals. Daphnet data set [89] offers more than eight hours of signals recorded with IMUs, from individuals with Parkinson’s disease. The authors also provide annotations about the start- and end-time of a specific type of event (“gait freeze”).

Similarly, HuGaDB database [90] contains ten hours of signals from IMUs and electromyography sensors. Here, 18 individuals perform activities such as walking or going upstairs. Similarly, in Ref. [91], there is 3-h monitoring of 27 individuals using the inertial sensor of a smartphone. However, the authors reported only the total number of footsteps per trial. Therefore, there are neither sufficient populations nor any information about the start/end of steps.

Therefore, we [92] included the start- and end- timestamps of all footsteps recorded by the IMUs (> 40,000 in total). Overall, this new open dataset contains around 8.5-h of gait signals from 230 subjects, collected by the foot IMUs (foot-worn). It is the largest freely available dataset (population and footstep annotations). Other works have already used part of the dataset. This initiative will further enhance the research around step detection, step importance (clinically), and step characteristics per disease. The dataset has already been used in numerous articles in computer science [77, 79] and clinical research [88, 93]. Such a dataset could significantly contribute to the flourishment of the systematic development of algorithms for risk of fall evaluation with more refined information. The shallow and deep learning predictive models mentioned above could benefit from such datasets.

### Postural control and oculomotor behavior

Defective gaze behavior was found to be a significant contributor [94] to impaired posture control in the elderly [95] but also in patients who have Parkinson’s disease [96]. Inversely, fixations on a target presented to the subjects improved locomotion in patients with Multiple sclerosis [39]. Recent studies also reported associations between saccades and posture in healthy populations. For example, saccadic eye movements significantly decreased the body sway magnitude in children [97] and older adults [98]. These results highlight the importance of the parieto-temporal cortex, brainstem, superior colliculus, and cerebellum in motor control since they are involved in oculomotor and postural control [99, 100].

Attentional aspects of the eye-movement task’s execution also significantly affect postural performance. Especially in diseases with attentional deficits, both eye movements [101, 102] and postural control [103] deficits were highly correlated. The frontal cortex, which is connected to the parietal areas [104], may also play an essential role in the interaction between visual and postural systems [105]. However, there are not many studies investigating the predictive power of gaze behavior in fall prediction using machine learning. Ecological tasks enable researchers to study the executive control of gaze and have been used in several contexts in recent years [99]. A recent study [101] investigated the interrelation of oculomotor and postural control during ecological tasks (in a “smart” flat). Bargiotas et al. [41], using machine learning, reported that parameters from eye-tracking trajectories, saccades, and fixations during an ecological task reflect important aspects of the postural impairment in Radiation-induced leukoencephalopathy (RIL) patients.

Taghvaei et al. [106] utilized a shallow learning model proposing an algorithm for real-time prediction of falls based on the acquired visual data of a user with a walking assistive system from a depth sensor. They fitted an autoregressive-moving-average (ARMA) model on the time-series from walking data to forecast the upcoming states. Then, a hidden Markov model (HMM) based classifier is built on the top of the ARMA model to predict falling in the upcoming time blocks.

### Motor style

Balance maintenance and locomotion involve complex sensorimotor transformations that require several sensory inputs to be integrated and the coordination of multiple motor outputs [107]. The coordination of posture and movement relies on anticipatory and reactive postural control mechanisms, modulated by sensory input, which are influenced by learning and experience. The sensory versant of the perceptive motor style is based on the fact that various systems (visual, somatosensory, vestibular) coordinate to maintain postural control [108]. An efficient, flexible, context-dependent postural control requires a continuous adaptive weighting of this three-sensory information. It was previously shown that such a reweighting process differs considerably among individuals [109–112]. The motor versant of the perceptive motor style is linked to the musculoskeletal system’s high number of degrees of freedom. Thus, complex processes such as postural control or locomotion exhibit significant inter-individual variability, leading to the concept of personalized perceptive motor behavior. A motor action can be performed in several ways by different actuators. Concerning gait, it was recently shown that individuals could be distinguished based on the variability in gait patterns [113]. The sensory-motor transformations in humans also exhibit large intra- and inter- variability. That variability is a complex product of internal and external noises with the neural mechanisms to keep movement fluctuations under control [114–118]. Therefore, the elements of human perceptual-motor styles can be found in (1) inter-individual variations and (2) intra-individual consistency/variability of individuals’ sensory-motor control.

The first challenge is to define sufficiently the elements of such style. We recently performed a walking/running protocol in healthy individuals using 3D motion tracking systems (CODA) [40], highlighting that there are elements of style in these simple activities. Following that, any changes in perceptive motor style (or comfort zone) open the question of at what point individual strategies were readjusted to maintain an optimal motor control (see [119] concerning walking and running economy) or if they reveal the onset of a pathological process. Such analysis can also help to follow individual’s recovery (see Ref. [120] on the thresholds to pathological variability during standing and walking).

## Conclusion and future challenges

Prevention and precision medicine, detection of frailty, and prediction of falls rely on quantifying normal and pathological human behavior, the perceptual-motor style of a given person, and its longitudinal follow-up. This style implies describing the action of 570 muscles acting on 200 joints in ecological conditions, i.e., at home, in the workplace, in a hospital. Disentangling the interconnection of these actions is not simple. This article described the efforts and achievements that have been made recently in frailty detection, especially in older adults who are more likely to experience recurrent falls. We describe recent trends, limitations, and challenges (analytics, sensors, and acquisition protocols) for frail detection and fall prediction systems. Finally, our article showed the beneficial and refreshing effect that machine learning and modern sensors (wearables and non-wearables) have on this topic.

It is essential to mention that the utility of posture or gait (or both) examination for fall prediction is depended on the context and the set objectives. Posture and gait are not examined on the same scale. Posturographic works usually search for signal fluctuation in a cm or even mm scale when gait analysis and spatiotemporal variables are usually in a cm or even m scale. Different phenomena are examined on different scales and sensors.

### Comparisons and analytic choices

As a result of the multiplication of available sensors, there is an explosion in the number of parameters available for clinical research (various biomarkers, multiple modalities, or parameters) for a given cohort. Although beneficial and rich, this fact challenges the data analysis via traditional approaches usually met in clinical research (such as T-statistics and linear regressions). Researchers can always perform more traditional statistics (multiple univariate regressions or hypothesis tests). They offer multiple advantages such as (a) the simplicity of the analysis, (b) the power of the result when clear evidence is present, and (c) the explainability/interpretability of the result. However, their results do not offer “predictability” rather than a statistical state of the current dataset (assuming that the dataset represents absolutely the group of investigation).

Moreover, when modest evidence is found in relatively small populations, the false-positive probability is significantly high. The level of that risk could be controlled when some criteria are met (see Ref. [42]) considering the quality of the study, the quality of the dataset, and the clinical strength of pre-set hypotheses). In more exploratory studies, though, some of the *p* values around 0.05, whichever side they may lie on, would be considered “interesting hints”, whereas concluding without thoughtful consideration from such findings should be generally avoided [43].

The multivariate and cross-validated approaches can decrease the uncertainty mentioned above to evaluate the risk of fall. Furthermore, the machine learning methodologies, and especially the well-established shallow learning methodologies such as decision trees, ensemble methods (random forest, boosting algorithms, etc.), logistic regression, Naive Bayes or support vector machines, offer a number of advantages such as:

- High-dimensionality: They can process highly dimensional and multimodal datasets.
- Non-linearity: They can easily handle possible non-linear associations (when asked) between symptoms and risk of fall evaluations
- Variables Interactions: they consider possible interactions between predictor variables to evaluate the actual contribution of every aspect to the risk of fall assessment.
- Missing values: They can handle missing values (usual in high dimensional datasets)
- Accurate indication of prospective predictability: They can provide (after cross-validation) an accurate indication of how the created model (and the relevant information) would perform in a new unseen dataset.
- Interpretability: They can usually provide interpretable results through the predictor importance of well-established variables.

Since they come from the same “family”, deep learning offers almost all the above advantages. Additionally, they usually come with an inherent feature engineering process, extracting and learning automatically better representations of raw data. Ideally, they need relatively large cohorts to “shine” (which is not usually the case in the biomedical field), and they usually show better performance than shallow learning methods. However, for higher predictability, they sacrifice explainability/interpretability. Unfortunately, it is not trivial to track what a deep neural network learned from the raw data before deciding about an individual’s risk of fall. This fact is usually seen as a disadvantage in most clinical practices. In some cases, though, deep learning can use raw data from extensive monitoring (from IMUs, smartwatches, smartphones, etc.) and work as a primary risk of fall screening. Such systems would alert experts to pay attention to specific individuals or even call them back to secured environments for further examinations.

To sum up, shallow learning methods, an especially Random Forest, offer a good compromise between performance, predictability, and explainability/interpretability. It seems that the current fall prediction literature (especially with non-wearables) is based on these methods. However, the advancements in monitoring systems and wearables facilitate further the creation of vast databases and cohorts. Moreover, the explainability of deep learning methodologies is getting more and more attention from machine learning researchers [121–123], which will hopefully provide more comprehensible solutions for non-experts. Therefore, these trends progressively create a friendlier set-up for more extended use of deep learning for the assessment of risk of fall (in the laboratory and “in the field”) in the future. A summary of the advantages and disadvantages of every analytic category is provided in Table 1. Table 1 presents the general aspects of the analytic categories that should be seen as general guidelines and by no means absolutely representative of every approach that is part of these categories. Every particular algorithm (in these categories) or statistical analysis has its own strengths and weaknesses.

**Table 1:** Summarizes the general advantages and disadvantages of the three analytic categories

Analytic approaches	Advantages	Disadvantages
Conventional statistics approaches	- Extremely fast - Easy to use - Interpretable (especially for linear phenomena) - Ideal for low dimensional datasets - Powerful in small cohorts	- Not accurate indication of prospective predictability (unless very large datasets are involved) - Interactions between predictors are usually not taken into account - Not ideal for modest evidence combined with high dimensions (weak predictors) - Increase of false-positive probability *without* *p* value adjustment - Increase of false-negative probability *with* *p* value adjustment
Shallow learning approaches	- Fast and effective - Ideal for low and high dimensional dataset - Ideal when there are missing values - Accurate indication of prospective predictability (with cross-validation schemes) - Ideal for non-linear associations - Take into account possible interactions between predictor variables - Ideal for medium and large cohorts - Interpretable (in many cases)	- Need some expertise - Sometimes computationally expensive to train - Not always fully interpretable
Deep learning approaches	- Not fast to be trained *but* very effective - Ideal for large datasets and time-series (e.g. raw signals from wearables) - Ideal for real time risk estimation (if the model is already trained), - Accurate indication of prospective predictability (with cross-validation schemes) - Handles non-linear associations with falls - Takes into account possible interactions between predictor variables - Ideal for large cohorts - Inherent feature engineering	- Need high expertise - Computationally expensive to train - Not easily interpretable

s

### Perspectives

The methodological advancements above create an important dynamic in a clinical setting as well as in an “ecological” setting. The future question is which of these two possibilities (or better combination) provides the best prediction of future falls. Clinical settings and laboratory-based acquisitions can gain performance when combined with ecological tasks. Recent meta-analytic efforts confirm the above arguments. Job et al. [124] examined evidence from selected works, which were subcategorized into (i) correlations between ecological and clinical measures and comparative statistics of (ii) prospective fall prediction and (iii) fall risk identification. There were many correlations between single ecological gait assessments and multiple clinical fall risk evaluations. The review, therefore, suggested that sensor-based assessments of gait in an ecological setting could significantly increase the prediction performance of clinical tests related to risk-of-fall evaluation.

Nevertheless, the authors stated that future studies are needed to understand what ecological features of gait should be considered, and standardize further the models’ definitions. Nouredanesh et al. [125] examined various studies in which inertial sensors were the only wearable system employed for fall risk assessment methods (FRAs) “in the field”. Gait, sitting, standing, lying, transitions, and gait events, such as turns and missteps, was explored. Many free-living fall predictors (FLFPs), e.g., the number of daily steps, were extracted from activity bouts and events. However, when FLFPs were further categorized into discrete domains defined by conceptual or data-driven models, the heterogeneity within the reviewed studies led to different results for similar FLFPs, limiting the ability to interpret and compare the evidence.

We are convinced that in the future, the majority of the systems developed for predicting falls in elderly or ambulatory persons should be tested in the natural environment. In particular, fall prevention applications that rely on gait may vary from surface to surface (standard floor uneven ground, stairs, sand, etc.). Users will be wearing the sensor-based solution for extended intervals, which makes the design of a user-friendly system a real challenge. A hybrid approach of wearable and ambient devices under reasonable cost would be beneficial to deal with obtrusive factors. Energy Efficiency will be a prerequisite: therefore, energy efficiency algorithms are required. Optimal sensor placement should be the rule because fixing sensors on various body parts can obtain various data types and extract various gait features. For example, sensors attached to thighs can monitor the process of sit-to-stand. The datasets are mainly small and consist of healthy subjects. Therefore, it will be essential to generate larger datasets, especially from the elderly. In that regard, the final acceptance of any system by the actual user is strongly associated with the level of integration of future users’ remarks at the initial stage of development. Also, the existing systems are not often in line with the patient confidentiality standards and regulations. The community has already started to set the foundations and the principles of such transition. We refer the interested reader to the excellent recent reviews [126–129]. In summary, the fusion of wearable and ambient sensors and a hybrid approach of proper education, IoT techniques, and clinical support is expected to affect the fall prediction and detection systems positively.

Fortunately, three revolutions in methodology have recently taken place in parallel with the development of the 5G: Cheap genotyping (genomics), Cheap biology (metabolomics), Behavioral quantification through cheap non-invasive sensors (i.e. humanomics, internet of things, internet of behavior). Such integrations would make it possible to compile exhaustive databases on Human behavior relative to falls. But, technically speaking, to collect databases on human behavior, it is necessary:

- To collect in the field and store quantitative data that continuously define human behavior in real-time (Ethomics). These databases must also be enriched with other data types such as clinical, psychological, and sociological data. It is a work of assembler with a requirement: raw, clean, and annotated databases. It is a job for an engineer.
- Using these databases, we can carry out the longitudinal follow-up of “high maintenance cohorts” (patients as well as military personnel, high-level athletes, etc.). The challenge here is not to “be buried” under petabytes of data. It is a job for a computer scientist
- To predict functional anomalies or pathologies thanks to these databases. Unfortunately, it is not obvious how to merge behavioral data and extract predictions. It is a job for mathematicians, physicians, and psychologists.

We tried to fulfill these objectives during the past few years. We learned in that process the necessity to associate all disciplines from the beginning of the project. We learned that in terms of targeted interventions, averaging populations is valuable but relatively limited compared to personalized follow-up. Above all, we learned to be patient because recording raw, clean, and indexed data in the field is more complex than in the lab.

## Footnotes

1 https://www.mobilise-d.eu/results-and-publications

## References

[1] Allen NE, Schwarzel AK, Canning CG (2013). Recurrent falls in Parkinson’s disease: a systematic review. Parkinsons Dis.

[2] Fernando E, Fraser M, Hendriksen J (2017). Risk factors associated with falls in older adults with dementia: a systematic review. Physiother Canada.

[3] Tinetti ME (2003). Clinical practice. Preventing falls in elderly persons. N Engl J Med.

[4] Melzer I, Benjuya N, Kaplanski J (2004). Postural stability in the elderly: a comparison between fallers and non-fallers. Age Ageing.

[5] Bergen G, Stevens MR, Burns ER (2016). Falls and fall injuries among adults aged ≥65 years—United States, 2014. MMWR Morb Mortal Wkly Rep.

[6] Scheffer AC, Schuurmans MJ, van Dijk N (2008). Fear of falling: measurement strategy, prevalence, risk factors and consequences among older persons. Age Ageing.

[7] Sylliaas H, Selbæk G, Bergland A (2012). Do behavioral disturbances predict falls among nursing home residents?. Aging Clin Exp Res.

[8] Schniepp R, Huppert A, Decker J (2021). Fall prediction in neurological gait disorders: differential contributions from clinical assessment, gait analysis, and daily-life mobility monitoring. J Neurol.

[9] Jahn K, Zwergal A, Schniepp R (2010). Gait disturbances in old age: classification, diagnosis, and treatment from a neurological perspective. Dtsch Arztebl Int.

[10] Maki BE, Zecevic A, Bateni H (2001). Cognitive demands of executing postural reactions: does aging impede attention switching?. NeuroReport.

[11] Zwergal A, Linn J, Xiong G (2012). Aging of human supraspinal locomotor and postural control in fMRI. Neurobiol Aging.

[12] Menant JC, Schoene D, Sarofim M, Lord SR (2014). Single and dual task tests of gait speed are equivalent in the prediction of falls in older people: a systematic review and meta-analysis. Ageing Res Rev.

[13] Kearney FC, Harwood RH, Gladman JRF (2013). The relationship between executive function and falls and gait abnormalities in older adults: a systematic review. Dement Geriatr Cogn Disord.

[14] Pettersson AF, Olsson E, Wahlund L-O (2005). Motor function in subjects with mild cognitive impairment and early Alzheimer’s disease. Dement Geriatr Cogn Disord.

[15] Dieterich M, Brandt T (2019). Perception of verticality and vestibular disorders of balance and falls. Front Neurol.

[16] Ganz DA, Bao Y, Shekelle PG, Rubenstein LZ (2007). Will my patient fall?. JAMA.

[17] Studenski S, Perera S, Patel K (2011). Gait speed and survival in older adults. JAMA.

[18] El-Khoury F, Cassou B, Charles M-A, Dargent-Molina P (2013) The effect of fall prevention exercise programmes on fall induced injuries in community dwelling older adults: systematic review and meta-analysis of randomised controlled trials. BMJ 347 10.1136/bmj.f6234 PMC3812467 24169944

[19] da Costa BR, Rutjes AWS, Mendy A (2012). Can falls risk prediction tools correctly identify fall-prone elderly rehabilitation inpatients? A systematic review and meta-analysis. PLoS ONE.

[20] Vassallo M, Poynter L, Sharma JC (2008). Fall risk-assessment tools compared with clinical judgment: an evaluation in a rehabilitation ward. Age Ageing.

[21] Omaña H, Bezaire K, Brady K (2021). Functional reach test, single-leg stance test, and tinetti performance-oriented mobility assessment for the prediction of falls in older adults: a systematic review. Phys Ther.

[22] Lusardi MM, Fritz S, Middleton A (2017). Determining risk of falls in community dwelling older adults: a systematic review and meta-analysis using posttest probability. J Geriatr Phys Ther.

[23] Barry E, Galvin R, Keogh C (2014). Is the Timed Up and Go test a useful predictor of risk of falls in community dwelling older adults: a systematic review and meta- analysis. BMC Geriatr.

[24] Quijoux F, Vienne-Jumeau A, Bertin-Hugault F (2020). Center of pressure displacement characteristics differentiate fall risk in older people: a systematic review with meta-analysis. Ageing Res Rev.

[25] Quijoux F, Nicolaï A, Chairi I (2021). A review of center of pressure (COP) variables to quantify standing balance in elderly people: algorithms and open-access code. Physiol Rep.

[26] Cortés OL, Piñeros H, Aya PA (2021). Systematic review and meta-analysis of clinical trials: In–hospital use of sensors for prevention of falls. Medicine (Baltimore).

[27] Ferreira RN, Ribeiro NF, Santos CP (2022). Fall risk assessment using wearable sensors: a narrative review. Sensors.

[28] Hemmatpour M, Ferrero R, Montrucchio B, Rebaudengo M (2019). A review on fall prediction and prevention system for personal devices: evaluation and experimental results. Adv Human-computer Interact.

[29] Montesinos L, Castaldo R, Pecchia L (2018). Wearable inertial sensors for fall risk assessment and prediction in older adults: a systematic review and meta-analysis. IEEE Trans Neural Syst Rehabil Eng.

[30] Sun R, Sosnoff JJ (2018). Novel sensing technology in fall risk assessment in older adults: a systematic review. BMC Geriatr.

[31] Balasubramanian CK (2015). The Community balance and mobility scale alleviates the ceiling effects observed in the currently used gait and balance assessments for the community-dwelling older adults. J Geriatr Phys Ther.

[32] Mancini M, Horak FB (2010). The relevance of clinical balance assessment tools to differentiate balance deficits. Eur J Phys Rehabil Med.

[33] Ruhe A, Fejer R, Walker B (2010). The test–retest reliability of centre of pressure measures in bipedal static task conditions—a systematic review of the literature. Gait Posture.

[34] de Sá FA, Junqueira Ferraz Baracat P (2014). Test–retest reliability for assessment of postural stability using center of pressure spatial patterns of three-dimensional statokinesigrams in young health participants. J Biomech.

[35] Duarte M, Freitas S, Zatsiorsky V (2011). Control of equilibrium in humans—Sway over sway.

[36] Ancona S, Faraci FD, Khatab E (2021). Wearables in the home-based assessment of abnormal movements in Parkinson’s disease: a systematic review of the literature. J Neurol.

[37] Doheny EP, Walsh C, Foran T (2013). Falls classification using tri-axial accelerometers during the five-times-sit-to-stand test. Gait Posture.

[38] Vienne A, Barrois RP, Buffat S (2017). Inertial sensors to assess gait quality in patients with neurological disorders: a systematic review of technical and analytical challenges. Front Psychol.

[39] Vienne A, Moreau A, Mantilla J (2017). Gaze constraint while walking in progressive multiple sclerosis: a feasibility study. Neurophysiol Clin.

[40] Mantilla J, Wang D, Bargiotas I (2020). Motor style at rest and during locomotion in humans. J Neurophysiol.

[41] Bargiotas I, Moreau A, Vienne A (2018). Balance impairment in radiation induced leukoencephalopathy patients is coupled with altered visual attention in natural tasks. Front Neurol.

[42] Feise RJ (2002). Do multiple outcome measures require p-value adjustment?. BMC Med Res Methodol.

[43] Wood J, Freemantle N, King M, Nazareth I (2014). Trap of trends to statistical significance: likelihood of near significant P value becoming more significant with extra data. BMJ.

[44] Bourke AK, van de Ven P, Gamble M (2010). Evaluation of waist-mounted tri-axial accelerometer based fall-detection algorithms during scripted and continuous unscripted activities. J Biomech.

[45] Massie S, Forbes G, Craw S et al (2018) Fitsense: employing multi-modal sensors in smart homes to predict falls. In: International conference on case-based reasoning. Springer, pp 249–263

[46] Kiprijanovska I, Gjoreski H, Gams M (2020). Detection of gait abnormalities for fall risk assessment using wrist-worn inertial sensors and deep learning. Sensors.

[47] Audiffren J, Bargiotas I, Vayatis N (2016). A non linear scoring approach for evaluating balance: classification of elderly as fallers and non-fallers. PLoS ONE.

[48] Bargiotas I, Kalogeratos A, Limnios M (2021). Revealing posturographic profile of patients with Parkinsonian syndromes through a novel hypothesis testing framework based on machine learning. PLoS ONE.

[49] Bargiotas I, Audiffren J, Vayatis N (2018). On the importance of local dynamics in statokinesigram: a multivariate approach for postural control evaluation in elderly. PLoS ONE.

[50] Speiser JL, Callahan KE, Houston DK (2021). Machine learning in aging: an example of developing prediction models for serious fall injury in older adults. J Gerontol Ser A.

[51] Tinetti ME (1986). Performance-oriented assessment of mobility problems in elderly patients. J Am Geriatr Soc.

[52] Shumway-Cook A, Brauer S, Woollacott M (2000). Predicting the probability for falls in community-dwelling older adults using the Timed Up & Go Test. Phys Ther.

[53] Perell KL, Nelson A, Goldman RL (2001). Fall risk assessment measures: an analytic review. J Gerontol Ser A Biol Sci Med Sci.

[54] Beauchet O, Fantino B, Allali G (2011). Timed up and go test and risk of falls in older adults: a systematic review. J Nutr Health Aging.

[55] Blum L, Korner-Bitensky N (2008). Usefulness of the berg balance scale in stroke rehabilitation: a systematic review. Phys Ther.

[56] Nicolai A, Audiffren J (2018) Model-space regularization and fully interpretable algorithms for postural control quantification. In: 2018 IEEE 42nd annual computer software and applications conference (COMPSAC). IEEE, pp 177–182

[57] Bargiotas I, Moreau A, Vayatis N, Ricard D (2019) Predicting future falls of parkinsonians using posturography and Random Forest. In: 2019 41th annual international conference of the IEEE engineering in medicine and biology society. IEEE, Berlin

[58] Bargiotas I, Audiffren J, Vayatis N (2019). Local assessment of statokinesigram dynamics in time: an in-depth look at the scoring algorithm. Image Process Line.

[59] Bargiotas I, Kalogeratos A, Limnios M et al (2020) Multivariate two-sample hypothesis testing through AUC maximization for biomedical applications. In: 11th hellenic conference on artificial intelligence, pp 56–59

[60] Sun R, Hsieh KL, Sosnoff JJ (2019). Fall risk prediction in multiple sclerosis using postural sway measures: a machine learning approach. Sci Rep.

[61] Clémençon S, Depecker M, Vayatis N (2013). Ranking forests. J Mach Learn Res.

[62] Eichler N, Raz S, Toledano-Shubi A (2022). Automatic and efficient fall risk assessment based on machine learning. Sensors.

[63] Liu C-L, Lee C-H, Lin P-M (2010). A fall detection system using k-nearest neighbor classifier. Expert Syst Appl.

[64] Breiman L (2001). Random forests. Mach Learn.

[65] Chagdes J, Rietdyk S, Haddad J (2009). Multiple timescales in postural dynamics associated with vision and a secondary task are revealed by wavelet analysis. Exp Brain Res.

[66] Savadkoohi M, Oladunni T, Thompson LA (2021). Deep neural networks for human’s fall-risk prediction using force-plate time series signal. Expert Syst Appl.

[67] Nicolai A, Limnios M, Trouve A, Audiffren J (2021). A langevin-based model with moving posturographic target to quantify postural control. IEEE Trans Neural Syst Rehabil Eng.

[68] Podsiadlo D, Richardson S (1991). The Timed “Up & Go”: a test of basic functional mobility for frail elderly persons. J Am Geriatr Soc.

[69] Pettersson B, Nordin E, Ramnemark A, Lundin-Olsson L (2020). Neither Timed Up and Go test nor Short Physical Performance Battery predict future falls among independent adults aged ≥75 years living in the community. J Frailty Sarcopenia Falls.

[70] de Morton NA, Berlowitz DJ, Keating JL (2008). A systematic review of mobility instruments and their measurement properties for older acute medical patients. Health Qual Life Outcomes.

[71] Vienne-Jumeau A, Quijoux F, Vidal P-P, Ricard D (2020). Wearable inertial sensors provide reliable biomarkers of disease severity in multiple sclerosis: a systematic review and meta-analysis. Ann Phys Rehabil Med.

[72] Vienne-Jumeau A, Oudre L, Moreau A (2020). Personalized template-based step detection from inertial measurement units signals in multiple sclerosis. Front Neurol.

[73] Dadashi F, Mariani B, Rochat S (2013). Gait and foot clearance parameters obtained using shoe-worn inertial sensors in a large-population sample of older adults. Sensors.

[74] Dibble LE, Nicholson DE, Shultz B (2004). Sensory cueing effects on maximal speed gait initiation in persons with Parkinson’s disease and healthy elders. Gait Posture.

[75] Glaister BC, Bernatz GC, Klute GK, Orendurff MS (2007). Video task analysis of turning during activities of daily living. Gait Posture.

[76] Rampp A, Barth J, Schuelein S (2015). Inertial sensor-based stride parameter calculation from gait sequences in geriatric patients. IEEE Trans Biomed Eng.

[77] Dot T, Quijoux F, Oudre L (2020). Non-linear template-based approach for the study of locomotion. Sensors.

[78] Mantilla J, Oudre L, Barrois R et al (2017) Template-DTW based on inertial signals: preliminary results for step characterization. In: 2017 39th annual international conference of the IEEE engineering in medicine and biology society (EMBC). IEEE, pp 2267–2270 10.1109/EMBC.2017.8037307 29060349

[79] Oudre L, Barrois-Müller R, Moreau T (2018). Template-based step detection with inertial measurement units. Sensors.

[80] Vienne-Jumeau A, Oudre L, Moreau A (2019). Comparing Gait Trials with Greedy Template Matching. Sensors.

[81] Zhou Y, Zia Ur Rehman R, Hansen C (2020). Classification of neurological patients to identify fallers based on spatial-temporal gait characteristics measured by a wearable device. Sensors.

[82] Kumar VC V, Ha S, Sawicki G, Liu CK (2020) Learning a control policy for fall prevention on an assistive walking device. In: 2020 IEEE international conference on robotics and automation (ICRA). IEEE, pp 4833–4840

[83] Hsieh C-Y, Shi W-T, Huang H-Y et al (2018) Machine learning-based fall characteristics monitoring system for strategic plan of falls prevention. In: 2018 IEEE international conference on applied system invention (ICASI). IEEE, pp 818–821

[84] Noh B, Youm C, Goh E (2021). XGBoost based machine learning approach to predict the risk of fall in older adults using gait outcomes. Sci Rep.

[85] Ye C, Li J, Hao S (2020). Identification of elders at higher risk for fall with statewide electronic health records and a machine learning algorithm. Int J Med Inform.

[86] Nait Aicha A, Englebienne G, Van Schooten KS (2018). Deep learning to predict falls in older adults based on daily-life trunk accelerometry. Sensors.

[87] Tunca C, Salur G, Ersoy C (2020). Deep learning for fall risk assessment with inertial sensors: utilizing domain knowledge in spatio-temporal gait parameters. IEEE J Biomed Heal Informatics.

[88] Barrois RP-M, Ricard D, Oudre L (2017). Observational study of 180° turning strategies using inertial measurement units and fall risk in poststroke hemiparetic patients. Front Neurol.

[89] Bachlin M, Plotnik M, Roggen D (2010). Wearable assistant for Parkinson’s disease patients with the freezing of gait symptom. IEEE Trans Inf Technol Biomed.

[90] Chereshnev R, Kertész-Farkas A (2018) HuGaDB: Human Gait Database for Activity Recognition from Wearable Inertial Sensor Networks. pp 131–141

[91] Brajdic A, Harle R (2013) Walk detection and step counting on unconstrained smartphones. In: Proceedings of the 2013 ACM international joint conference on pervasive and ubiquitous computing. ACM, New York, NY, USA, pp 225–234

[92] Truong C, Barrois-Müller R, Moreau T (2019). A data set for the study of human locomotion with inertial measurements units. Image Process Line.

[93] Barrois R, Gregory T, Oudre L (2016). An automated recording method in clinical consultation to rate the limp in lower limb osteoarthritis. PLoS ONE.

[94] Ewenczyk C, Mesmoudi S, Gallea C (2017). Antisaccades in Parkinson disease: a new marker of postural control?. Neurology.

[95] Chapman GJ, Hollands MA (2006). Evidence for a link between changes to gaze behaviour and risk of falling in older adults during adaptive locomotion. Gait Posture.

[96] Vitório R, Gobbi LTB, Lirani-Silva E (2016). Synchrony of gaze and stepping patterns in people with Parkinson’s disease. Behav Brain Res.

[97] Ajrezo L, Wiener-Vacher S, Bucci MP (2013). Saccades improve postural control: a developmental study in normal children. PLoS ONE.

[98] Aguiar SA, Polastri PF, Godoi D (2015). Effects of saccadic eye movements on postural control in older adults. Psychol Neurosci.

[99] Leigh RJ, Zee DS (2015). The neurology of eye movements.

[100] Ouchi Y, Okada H, Yoshikawa E (1999). Brain activation during maintenance of standing postures in humans. Brain.

[101] Deubel H, Schneider WX (1996). Saccade target selection and object recognition: evidence for a common attentional mechanism. Vision Res.

[102] Rizzolatti G, Riggio L, Dascola I, Umiltá C (1987). Reorienting attention across the horizontal and vertical meridians: evidence in favor of a premotor theory of attention. Neuropsychologia.

[103] Belenkii VE, Gurfinkel VS, Paltsev EI (1967) On the control elements of voluntary movements. Biofizika 5623488

[104] Gaymard B, Lynch J, Ploner CJ (2003). The parieto-collicular pathway: anatomical location and contribution to saccade generation. Eur J Neurosci.

[105] Bonnet CT, Szaffarczyk S, Baudry S (2017). Functional synergy between postural and visual behaviors when performing a difficult precise visual task in upright stance. Cogn Sci.

[106] Taghvaei S, Jahanandish MH, Kosuge K (2017). Autoregressive-moving-average hidden Markov model for vision-based fall prediction—an application for walker robot. Assist Technol.

[107] Ting LH, McKay JL (2007). Neuromechanics of muscle synergies for posture and movement. Curr Opin Neurobiol.

[108] Merfeld DM, Zupan L, Peterka RJ (1999). Humans use internal models to estimate gravity and linear acceleration. Nature.

[109] Bonan IV, Gaillard F, Ponche ST (2015). Early post-stroke period: a privileged time for sensory re-weighting?. J Rehabil Med.

[110] Isableu B, Ohlmann T, Crémieux J, Amblard B (2003). Differential approach to strategies of segmental stabilisation in postural control. Exp Brain Res.

[111] Lacour M, Barthelemy J, Borel L (1997). Sensory strategies in human postural control before and after unilateral vestibular neurotomy. Exp Brain Res.

[112] Vibert N, MacDougall HG, De Waele C (2001). Variability in the control of head movements in seated humans: a link with whiplash injuries?. J Physiol.

[113] Sprager S, Juric MB (2015). Inertial sensor-based gait recognition: a review. Sensors.

[114] Kikkert LHJ, Vuillerme N, van Campen JP (2016). Walking ability to predict future cognitive decline in old adults: a scoping review. Ageing Res Rev.

[115] Mortaza N, Abu Osman NA, Mehdikhani N (2014). Are the spatio-temporal parameters of gait capable of distinguishing a faller from a non-faller elderly. Eur J Phys Rehabil Med.

[116] Dasenbrock L, Heinks A, Schwenk M, Bauer JM (2016). Technology-based measurements for screening, monitoring and preventing frailty. Z Gerontol Geriatr.

[117] Schwenk M, Howe C, Saleh A (2014). Frailty and technology: a systematic review of gait analysis in those with frailty. Gerontology.

[118] Dingwell JB, Cusumano JP (2015). Identifying stride-to-stride control strategies in human treadmill walking. PLoS ONE.

[119] Moore IS (2016). Is there an economical running technique? A review of modifiable biomechanical factors affecting running economy. Sport Med.

[120] König N, Taylor WR, Baumann CR (2016). Revealing the quality of movement: a meta-analysis review to quantify the thresholds to pathological variability during standing and walking. Neurosci Biobehav Rev.

[121] Lundberg SM, Lee S-I (2017) A unified approach to interpreting model predictions. Adv Neural Inf Process Syst 30

[122] Ribeiro MT, Singh S, Guestrin C (2016) “Why should i trust you?” Explaining the predictions of any classifier. In: Proceedings of the 22nd ACM SIGKDD international conference on knowledge discovery and data mining. pp 1135–1144

[123] Chen C, Li O, Tao C, Barnett AJ, Rudin C, Su JK (2019) This looks like that: deep learning for interpretable image recognition. Adv Neural Informat Process Syst 32

[124] Job M, Dottor A, Viceconti A, Testa M (2020). Ecological gait as a fall indicator in older adults: a systematic review. Gerontologist.

[125] Nouredanesh M, Godfrey A, Howcroft J (2021). Fall risk assessment in the wild: a critical examination of wearable sensor use in free-living conditions. Gait Posture.

[126] Rajagopalan R, Litvan I, Jung T-P (2017). Fall prediction and prevention systems: recent trends, challenges, and future research directions. Sensors.

[127] Zhao G, Chen L, Ning H (2021) Sensor-based fall risk assessment: a survey. In: Healthcare. multidisciplinary digital publishing institute, p 1448 10.3390/healthcare9111448 PMC8624006 34828494

[128] Usmani S, Saboor A, Haris M (2021). Latest research trends in fall detection and prevention using machine learning: a systematic review. Sensors.

[129] Tanwar R, Nandal N, Zamani M, Manaf AA (2022) Pathway of trends and technologies in fall detection: a systematic review. In: Healthcare. multidisciplinary digital publishing institute, p 172 10.3390/healthcare10010172 PMC8776012 35052335

